# Perception of short, but not long, time intervals is modality specific: EEG evidence using vibrotactile stimuli

**DOI:** 10.1093/cercor/bhaf051

**Published:** 2025-03-06

**Authors:** Nicola Thibault, Andréanne Sharp, Philippe Albouy, Simon Grondin

**Affiliations:** École de Psychologie, Faculté des sciences sociales, Pavillon Félix-Antoine-Savard, 2325 Rue des Bibliothèques, Université Laval, Québec G1V 0A6, Canada; CERVO Brain Research Centre, 2301 Av. D'Estimauville, Québec G1J 2G3, Canada; CERVO Brain Research Centre, 2301 Av. D'Estimauville, Québec G1J 2G3, Canada; Faculté de Médecine, Université Laval, Pavillon Ferdinand-Vandry, 1050 Av. de la Médecine, Québec G1V 0A6, Canada; École de Psychologie, Faculté des sciences sociales, Pavillon Félix-Antoine-Savard, 2325 Rue des Bibliothèques, Université Laval, Québec G1V 0A6, Canada; CERVO Brain Research Centre, 2301 Av. D'Estimauville, Québec G1J 2G3, Canada; Centre for Research on Brain, Language and Music, International Laboratory for Brain, Music and Sound Research, Pavillon Marie-Victorin, Université de Montréal, 90 Vincent D'Indy Ave, Outremont, Quebec H2V 2S9, Canada; École de Psychologie, Faculté des sciences sociales, Pavillon Félix-Antoine-Savard, 2325 Rue des Bibliothèques, Université Laval, Québec G1V 0A6, Canada; CERVO Brain Research Centre, 2301 Av. D'Estimauville, Québec G1J 2G3, Canada

**Keywords:** EEG, intervals, oddball, time perception, vibrotactile

## Abstract

A longstanding debate in cognitive neuroscience questions whether temporal processing is modality-specific or governed by a “central clock” mechanism. We propose that this debate stems from neglecting the duration of the intervals processed, as studies supporting modality-specific models of time perception often focus on below 1.2-s intervals. To address this, we examined the neuronal dynamics underlying the perception of time intervals shorter and longer than 1.2-s using vibrotactile stimuli. Twenty participants underwent electroencephalogram recordings during a passive tactile oddball paradigm. We compared brain responses to standard and deviant intervals, with deviants occurring either earlier or later than the standard in both below and above 1.2-s conditions. Event-related potentials revealed distinct deviance-related components: a P250 for deviance detection of short deviants and an N400 long deviants. Generators lied in a modality-specific network for short intervals, while long intervals activated a broader, higher-level network. We found no evidence of the contingent negative variation in the tactile modality, questioning its role as a universal marker of temporal accumulation. Our findings suggest that short intervals involve modality-specific circuits, while longer intervals engage distributed networks, shedding light on whether temporal processing is centralized or distributed.

## Introduction

The question of whether the processing of temporal information relies on a singular, central mechanism or on processes within sensory modalities remains an open question. Central clock models, such as the pacemaker models, have consistently been invoked to account for capacity to process temporal information (Gibbon 1977; Gibbon et al. 1984; Grondin 2001). The Scalar Expectancy Theory (SET) exemplifies this stance. In the SET, the internal clock is referred to as a pacemaker-counter device. It emits pulses that are accumulated in a counter and serves as the foundation for the estimation of time intervals. The SET posits that the variability-to-time ratio should remain constant. However, several psychophysical studies have shown that such is not the case (Grondin 2014, 2001; Grondin 2010). This ratio increases when intervals reach 1.2 to 1.5-s (Gibbon et al. 1997; Grondin 2014, 2012; Grondin et al. 2015). Given this rationale, some researchers propose a distinction between the processing of below 1.2-s and above 1.2-s intervals (Rammsayer and Lima 1991; Lewis and Miall 2003b; also labeled as sub-second and supra-second intervals, respectively).

Contrary to central clock models, there is an increasing body of evidence that supports the notion that time perception may instead rely on dedicated sensory-specific processes (Bueti 2011; Bueti et al. 2008a). According to this perspective, temporal information is embedded within a sensory modality’s circuitry. Several studies have demonstrated that participants exhibit varying levels of performance when asked to differentiate time intervals marked by either auditory or visual signals. Performances are consistently worse in the visual than in the auditory conditions (Wearden et al. 1998; Penney et al. 2000; Grondin 2003; Droit-Volet et al. 2007; Grondin and McAuley 2009). Consistent with dedicated sensory-specific theories, the processing of time intervals in these 2 modalities has distinct electrophysiological markers and generators (Chen et al. 2010). These processes require various levels of attentional control, with the visual modality typically requiring more attentional resources than the more salient auditory stimuli (Recanzone 2003; Menon and Uddin 2010; Birkett and Talcott 2012; Zélanti and Droit-Volet 2012).

Some neurophysiological findings also corroborate a perspective of time perception that is modality specific. Evidence from neuromodulation indicates that inhibiting the auditory cortex reduces the accuracy of auditory and visual time intervals, but inhibiting the visual cortex only affects visual time intervals (Bueti et al. 2008a; Kanai et al. 2011; Mioni et al. 2016). These results suggest that time processing may be sensory-specific, but also highlight the auditory cortex’s significant role in multi-sensory temporal processes.

Another set of psychophysical studies raises questions about the unique, central-clock perspective. We know that the discrimination of brief empty time intervals is much better with auditory than with visual or tactile markers (Azari et al. 2022). However, the discrimination of intervals is significantly better when the 2 successive signals surrounding an empty interval are both tactile or both visual (intramodal conditions) compared to when the interval is marked by successive signals presented using a combination of modalities, such as auditory, visual, or tactile (intermodal conditions; Grondin and Rousseau 1991; Azari et al. 2020; Azari et al. 2022). This general finding suggests that for brief intervals, processing could occur within modalities, in unimodal conditions, but for multisensory intervals, the processing could require the contribution of a central, more cognitively controlled process as is the case with longer intervals (Grondin and Rousseau 1991). Such a perspective is consistent with the idea that the perception of below 1.2-s (which we will describe as short) intervals is based on a sensory/automatic mode of processing (Lewis and Miall 2003a, 2003b; Thibault et al. 2023) while the estimation of above 1.2-s (which we will describe as long) intervals is based on the contribution of cognitive processes (Macar et al. 1994; Zakay and Block 1997; Brown 2006; Brown et al. 2013; Thibault et al. 2023; Thibault et al. 2024).

We propose that the debate opposing central clock models and sensory-specific models arises from not considering the length of to-be-encoded temporal intervals. The literature predominantly endorsing the sensory-specific branch of models uses below 1.2-s (short) intervals (Bosco et al. 2008; Bueti et al. 2008a; Bueti et al. 2008b; Kanai et al. 2011). Using an auditory timing oddball paradigm, Thibault et al. (2023 and 2024) showed that the primary auditory cortex was recruited only for processing shorter intervals, whereas for longer intervals, the activated network was more distributed, and less sensory-oriented. If the processing of short intervals is sensory specific, we should thus observe similar result profiles with the same paradigm delivered to another sensory modality. To our knowledge, no study has investigated the electrophysiological markers and generators of short and long time intervals in the tactile modality. We thus used tactile stimuli delivered to both hands via vibro-tactile gloves (Chauvette et al. 2024) and a passive oddball paradigm to contrast short and long timing processes to resolve the ongoing debate about the modality-specificity of time perception. If the processing of short intervals is sensory specific, they should be processed by sensory-specific circuitry (the somatosensory cortices in this experiment), as it was the case with our past experiments for the auditory modality (Thibault et al. 2023; Thibault et al. 2024). If the processing of long time intervals is more cognitively oriented, they should be processed by a more distributed, non-sensory specific, central-executive network (Thibault et al. 2023).

## Materials and methods

### Ethics

All procedures were approved by the Ethics Committee of the CIUSSS de la Capitale Nationale: 2021-2156. All procedures were carried out in accordance with relevant guidelines and regulations of the Ethics Committee of the CIUSSS de la Capitale Nationale: 2021-2156.

### Participants

Twenty healthy participants (13 females and 7 males; mean age, 25.20; SD, 2.14; age range; 21 to 31, 4 were pre-university students, 8 were bachelor students, 4 were master’s student, and 4 were doctorate students; 17 right-handed and 3 left-handed), all with self-reported normal hearing, no history of neurological or psychiatric disorders, gave their informed consent to participate in this study and received monetary compensation for their participation.

### Vibrotactile gloves and stimuli

Tactile stimulations were delivered through 2 vibrotactile gloves to both hands. The vibrating gloves were the same as in Chauvette et al. (2024). They are equipped with 6 independent audio-haptic voice-coil exciters per hand. The voice-coil transducers designed to deliver vibrotactile output (TEAX14C02-8 Compact Audio Exciter) had a diameter of 14 mm. Stimuli were sent via a12- channel class D audio MA1260 power amplifier (DaytonAudio, Springboro, OH, United States), linked via an audio cable to the software Presentation (Neurobehavioral systems, Albany, CA, United States) on a computer. All vibrations were 500 Hz and lasted 33 ms, with a 33-ms ascending-descending envelope, for a total of 100 ms.

### Task and procedure

The task and procedures are identical to Thibault et al. (2023 & 2024), except that vibrotactile instead of auditory stimuli were used to mark time intervals. Participants were scheduled for 2 sessions. The first was the MRI session, lasting approximately 50 min with installation. The second was the electroencephalogram (EEG) session which lasted approximately 2 h and 15 min (1 h and 50 min of experimentation and approximately 25 min of EEG preparation and set-up). During the EEG session, participants went through twelve 8-min blocks of an oddball paradigm with time intervals.

After installation of the EEG system, we tested whether participants could hear the vibrotactile gloves to control for auditory perception. Half of 1 block (5 min) was presented to the participants. In this control block, the gloves were hanging beside the participants (without tactile stimulation) approximately where they were located during the experimental block. This session verified whether participants could hear the noises produced by the vibration of the speakers on the gloves. This attests that no auditory responses were associated to the presentation of vibrotactile stimuli. Furthermore, a white noise was presented continuously with an Audiotechnica ATH-M50x headset during the main experiment. The volume of the white noise was adjusted so that the participant could not hear the sound produced by the gloves, similarly to the methodology used in previous study (Sharp et al. 2019; Sharp et al. 2020; Chauvette et al. 2024).

The EEG session consisted of 12 blocks. Each block was separated by a 3-min break. The task consisted in a passive perception of a tactile oddball paradigm; eyes opened with a fixation cross displayed on the screen. For each block, 40 trials of 10 time intervals (marked by vibrotactile stimuli) were presented for each condition (short and long). It is relevant to note that all trials were presented continuously without inter-trial intervals, which results in a continuous flow of stimuli (corresponding to a classic oddball paradigm). For each trial, a deviant empty time interval appearing between vibrations was pseudo-randomly presented across the standard empty time intervals. The standards and deviants are referring to empty time intervals presented. In a series of 10 empty time intervals, 9 empty time intervals were “standards” (which corresponded to 0.8 s in the short condition, and 1.6 s in the long condition) and 1 was “deviant” (the empty time interval was either shorter/early) or longer/delayed). There was a repetition of at least 4 standard time intervals before a deviant time interval was presented. For half of the blocks, the short condition trials were presented first, and for the other half, the long condition trials were presented first. The deviant empty time interval for each sequence of 10 empty time intervals was selected randomly. The deviant empty time intervals were either early (short: 0.64, 0.72 s; long: 1.28, 1.44 s) or delayed (short: 0.88, 0.96 s; long: 1.76, 1.92 s) as compared to the empty standard interval. Empty time intervals, by opposition to filled intervals, were used to avoid tactile fatigue. Furthermore, for the range of durations used in the current experiment, empty intervals are at least as easily discriminated as filled intervals (Grondin 1993; Grondin et al. 1998). This resulted in 60 deviants for each condition. Sixty standard traces were randomly selected between all pre-deviant standards to match the number of deviants for comparison. The participants did not have to make any judgments (no behavioral task) and were instructed to pay attention to the vibrations that would be presented. They were also instructed to not make contact with the chair or the desk with their gloves, reducing sound amplification.

Presentation software (Neurobehavioral Systems, Albany, CA, United States) was used for the delivery of the experimental protocol and to trigger tactile stimuli. For the twelve blocks, the short condition and the long condition were presented in alternation, switching the sequence for the following block. Somatosensory event-related potentials (ERP) for each condition were then studied at the sensors and source levels.

### Anatomical data

All participants underwent a 3D anatomical MPRAGE T1-weighted Magnetic Resonance Imaging scan on a 3 T Siemens PRISMA (Siemens AG, Erlangen, Germany) before (no more than 1 wk) the EEG recording session. The anatomical volume consisted of 192 sagittal slices with 1 mm^3^ voxels (TR = 2,300 ms, TE = 298 ms), covering the whole brain. The scalp and cortical surfaces were extracted from the T1-weighted anatomical MRI. A surface triangulation was obtained for each envelope using the segmentation pipeline available in CAT12 (http://www.neuro.uni-jena.de/cat/) with SPM (Statistical Parametric Mapping software; http://www.fil.ion.ucl.ac.uk/spm/) and default parameter settings. The individual high-resolution cortical surfaces (about 75,000 vertices per surface) were down-sampled to 15,000 vertices using Brainstorm to serve as image supports for EEG source imaging.

### E‌EG recording

A 64-channel EEG cap with active electrodes (ActiCap—Brain Vision Solutions, Brain Products) was used to capture the electroencephalographic activity with 2 32-BrainAmp MR Plus amplifiers (Brain Products, Munich, Germany). The installation of the EEG was completed with respect to the standard 10 to 20 installation. The signal was band-pass filtered between DC and 1,000 Hz and digitized at a sampling rate of 1,000 Hz.

All channels were referenced with an electrode placed on the nose and with a forehead ground. All electrodes had an impedance of < 20 kΩ. EEG data were preprocessed using Brainstorm software (Tadel et al. 2011) combined with Fieldtrip functions (http://www.fieldtriptoolbox.org/) and MATLAB (MathWorks, https://www.mathworks.com/products/matlab.html). The EEG preprocessing included notch filtering of the wall outlets’ contamination (removed the 60, 120, and 180 Hz). A band-pass filter for frequencies of interest (ERPs) between 1 and 16 Hz was applied for the ERP preprocessing. The filtered data were subjected to independent component analysis (ICA) using EEGlab functions (https://sccn.ucsd.edu/eeglab/). ICA removes muscle artifacts such as blinking and eye movements. Using time-course and topographic information, components representing clear ocular artifacts were identified and removed from the filtered data. Each event (deviant and standard) was inspected from −1,900 to 1,900 ms relative to the onset of each sound, and trials for which the signal varied by more or less than 150 μV during the duration of the trial were excluded: between 17 and 62 trials per condition were kept for each participant. For each epoch, a baseline correction of 100 ms (−100 to 0 ms) before the previous standard.

### Source modeling

Source reconstruction of the ERPs was performed using functions available in Brainstorm, all with default parameter settings (Tadel et al. 2011) as in Albouy et al. (Albouy et al. 2022) and Thibault et al. (2023). Forward modeling was performed using a realistic head model: Symmetric Boundary Element Method from the open-source software OpenMEEG. The lead-fields were computed from elementary current dipoles distributed perpendicularly to the cortical surface. EEG source imaging was performed by linearly applying Brainstorm’s weighted-minimum norm operator onto the preprocessed data. The data were previously projected away from the spatial components of artifact contaminants. For consistency between the projected data and the model of their generation by cortical sources, the forward operator was projected away from the same contaminants using the same projector as for the EEG data. The EEG data were projected on a cortical surface template available in Brainstorm (adult cortical surface of 15,002 vertices serving as image support for EEG source imaging). Baseline normalization (−100 to 0 ms relative to the previous standard onset) was calculated using a Z-score, and 3-mm Gaussian spatial smoothing was applied to the cortical mesh. ERPs were calculated separately for the standard and deviant conditions for each participant. Source reconstruction was performed for each participant for the entire ERPs time period. The ERPs at the sensor and source levels for each standard (short: 0.8 s; long: 1.6 s) and deviant type (early short: 0.64, 0.72 s; early long: 1.28, 1.44 s; delayed short: 0.88, 0.96 s; delayed long: 1.76, 1.92 s) of all participants were averaged. Only the source contrasts for the ERPs significant periods were calculated.

### Granger causality

The bivariate Granger causality values were calculated using algorithms implemented in Brainstorm (Tadel et al. 2011) as in Thibault et al. 2023. Granger causality is a measure of directed functional connectivity (Granger et al. 2000; Dhamala et al. 2008). The NxN Granger causality values were calculated with a model order of 2, using the maximum value of connectivity applied after the computation of connectivity measure. Bivariate Granger causality values were calculated for standards and deviants for the long early and delayed condition (300–500 ms) and for the short early condition (150 to 350 ms) for periods identified in Fig. 2. For the short early condition, a GC matrix was calculated for the left primary somatosensory cortex (SI), the left premotor cortex (PMC), the left supplementary motor area (SMA) and the left motor cortex. For the short delayed condition, no matrix was calculated because only 1 cluster existed. For the long early condition, bivariate GC values were computed for the bilateral inferior frontal gyrus (IFG), right anterior prefrontal cortex, right temporal pole, left motor cortex, right insula, and left PMC. For the delayed long condition, bivariate Granger causality values were calculated for the bilateral IFG, left SMA, right secondary auditory cortex, left PMC, bilateral SI, right parietal cortex, bilateral motor cortex, right insula, and right temporal pole. This resulted in a connectivity matrix for each subject by condition for deviants and standards.

### Statistics

Whole-brain analyses (sensors and sources of deviants vs. standard contrasts) of EEG activity were performed using non-parametric permutation testing and cluster randomization statistics in time and space (1,000 permutations, sensor cluster alpha = 0.05, sources cluster alpha short early = 0.025, sources cluster alpha long early and delayed = 0.020, k *≥* 30) as implemented in Fieldtrip (www.fieldtriptoolbox.org/). The standard interval traces were randomly selected among all pre-deviant standard. These analyses were done on the ERPs data for 3 time periods corresponding to the P100, P200, N400 ERPs components (Kutas and Federmeier 2011; Morozova et al. 2023). To account for potential changes in deviance detection over time or in subsequent responses to the deviants, we have also performed the same analysis but contrasting deviants to the standards presented immediately after the deviants. The same analyses (with cluster alpha = 0.05 for sensors) were performed for the CNV time-period (−500 to 0 ms). Moreover, paired non-parametric Wilcoxon tests were performed to contrast deviants against standards connectivity matrices per condition (short early, and long early and delayed) on the bivariate Granger causality values. False discovery rate (FDR) correction was applied to the Wilcoxon tests and an alpha of 0.05 was chosen. The result of this test advises on which regions are significantly interconnected and the direction of this connection while deviance occurs for each condition.

## Results

**Fig. 1:**
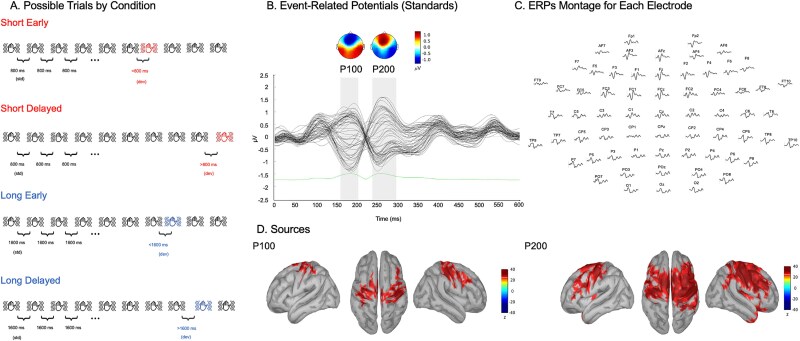
ERPs and sources for standard intervals and trial illustrations. A) Examples of possible trials for each condition. B) ERPs for standard intervals. The grand average of the signals (all electrodes) for a trial time window (0 to 600 ms) for the standard intervals across all participants and conditions (short and long). Topographies represent the bilateral biphasic somatosensory ERPs components, i.e. (P100 and P200), gray shading represent mean standard error). C) ERPs montage displaying ERPs for all electrodes (0 to 600 ms). d) Cortical surface renditions in the MNI space show bilateral generators in somatosensory regions (z-score relative to baseline −100 to 0 ms) for the bilateral biphasic periods.

**Fig. 2:**
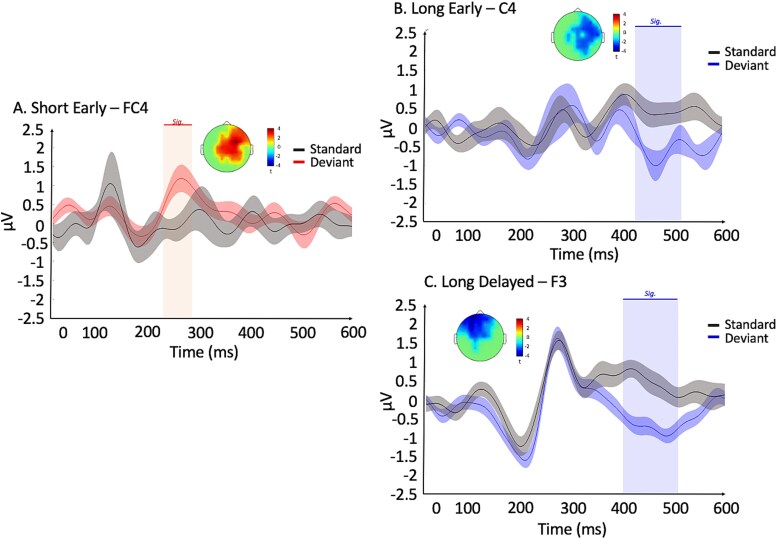
Deviants vs. standards at the sensor level: ERPs of standards (in black) and deviants for A) short early (deviants in red, FC4 electrode), B) long early (C4 electrode), and C) long delayed (deviants in blue, F3 electrode) conditions averaged across trials and participants. 0 ms corresponds to the onset of the vibrotactile stimulus either on time (standard) or delayed/early (deviant). Shadowed lines illustrate the standard error of the mean. Cluster-corrected topographies of significant differences are displayed for the significant effects. Blue and red vertical shadowing illustrate significant periods.

Participant were presented with a passive vibrotactile oddball paradigm, delivered to both hands, during an EEG recording. ERPs of all standard intervals averaged across participants showed biphasic somatosensory centro-parietal positivity (P100 component) and a fronto-central positive peak (somatosensory P200, see Fig. 1A and B; Morozova et al. 2023). Central electrodes were selected for illustration (see Fig. 2). However, note that all analyses reported below were conducted at the whole-brain level with corrections for multiple comparisons in time (samples) and space (electrodes/vertices) domains and were thus not performed solely on electrodes of interest. Source reconstructions of the time windows centered on the peak of each component (source illustration in Fig. 1: P100, 190 ms; P200, 260 ms) were computed (z-scored with baseline activity). The generators consisted in, as expected, primary somatosensory cortex in shorter latency component (Hoechstetter et al. 2001), while later component had more distributed generators including the secondary somatosensory cortices and the motor cortex (Karhu and Tesche 1999; Francis et al. 2000; Fig. 1C).

As per Thibault et al. (2023)’s protocol, we first investigated whether a contingent negative variation (CNV) was elicited before deviance detection. We performed the CNV analyses for delayed deviants only, using cluster-corrected (in time and space) non-parametric permutation tests (1,000 permutations and cluster alpha = 0.05). In contrast to Thibault et al. (2023)’s results with auditory stimuli, there was no significant CNV for time perception with vibrotactile stimuli.

To examine whether deviants prompted differences in ERPs when compared to their respective standards, we investigated the brain responses associated with the onset of deviance detection for short- and long-time intervals after the onset of the vibrotactile stimulus, marking the beginning of the processing of the deviant interval. We performed non-parametric permutation tests with cluster-based correction in time and space to compare the deviant ERPs of the short and long conditions with their corresponding standard ERPs. Tests were performed for 3 periods defined in the ERPs for standard interval (Fig. 1): 100 to 200, 200 to 300, 400 to 500 ms from post-stimulus onset (Fig. 2). Significant differences between deviants and standards were observed for the short early condition around the P250 time window (*P* = 0.02, for 224 to 276 ms, Fig. 2A) and around the N400 period for long early and delayed conditions (early: *P* = 0.02, for 408 to 500 ms, Fig. 2B; delayed: *P* = 0.01, for 400 to 500 ms, Fig. 2C). No significant component was observed in the short delayed condition (*P* > 0.05). Similarly, no other significant component was observed in the other time windows of interest (all *P*s > 0.05). Cluster-corrected topographies were calculated for the long and short delayed deviants against their respective standards for the peak of the significant period. Statistical tests were corrected for time and space (cluster-corrected). Shown sensors in the following figures were selected as they were the maximum statistical values (based on T-values).

**Fig. 3:**
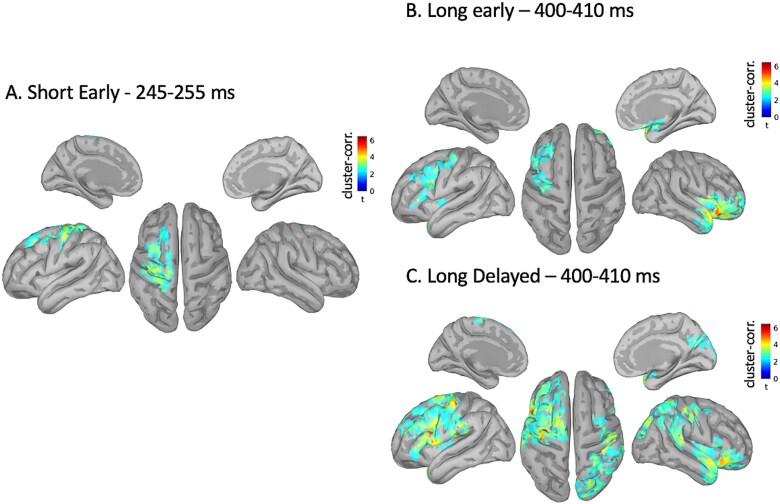
Deviants vs. standards at the source level: cluster-corrected source reconstructions (cluster alpha short early = 0.025, cluster alpha long early and delayed = 0.020, k ≥ 30) for the peak of the significant periods [A) short early, B) Long early, C) Long delayed] illustrated in Fig. 2.

The same analysis pipeline was conducted at the source level with cluster-based non-parametric permutation tests (alpha = 0.05 and 1,000 permutations, k ≥ 30) averaged over the peak periods identified at the sensor level (P250 for short: 245 to 255 ms; N400 for long: 400 to 410 ms). When deviance occurred in the short early condition, deviance detection-related activity was observed in the left primary SI, the left PMC, the left SMA and the left motor cortex (Fig. 3A). In the long early condition, deviance detection was observed for the bilateral IFG, right anterior prefrontal cortex, right temporal pole, left motor cortex, right insula, and left PMC (Fig. 3B). In the long delayed condition generators were the bilateral IFG, left SMA, right secondary auditory cortex, left PMC, bilateral SI, right parietal cortex, bilateral motor cortex, right insula, and right temporal pole (Fig. 3C).

**Fig. 4:**
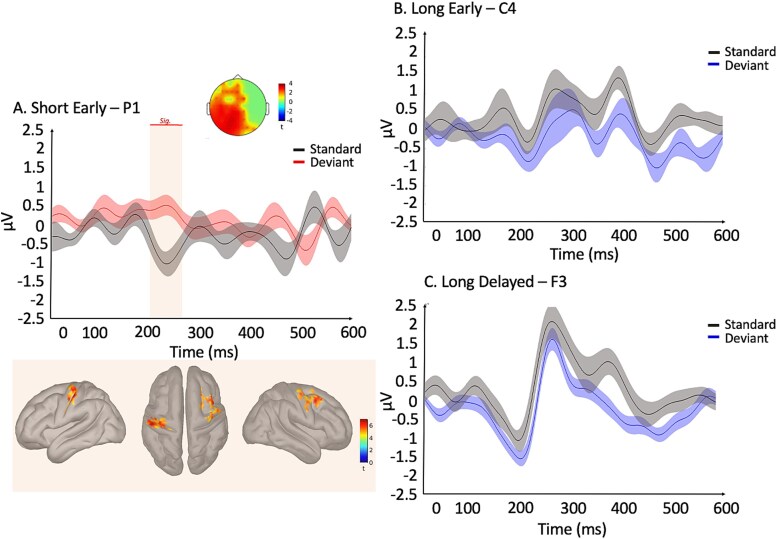
Deviants vs. post-deviant standards: ERPs of standards (in black) and deviants for A) short early (deviants in red, P1 electrode) with the component’s source generators below, B) long early (C4 electrode), and C) long delayed (deviants in blue, F3 electrode) conditions averaged across trials and participants. 0 ms corresponds to the onset of the vibrotactile stimulus either on time (standard) or delayed/early (deviant). Shadowed lines illustrate the standard error of the mean. Cluster-corrected topographies of significant differences are displayed for the significant effects. Blue and red vertical shadowing illustrate significant periods.

We then performed the same contrasts analyses on sensors and sources with post-deviant standards (instead of pre-deviant standards) to account for potential changes in deviance detection over time or in subsequent responses to the deviants (source reconstructions cluster alpha of 0.001). The sensor analyses (cluster-corrected in time and space) revealed a P250 component for the short early condition (*P* = 0.034, 200 to 274 ms; Fig. 4A) while no effect was observed for both the long early and delayed conditions for all periods (early: *P* > 0.172 and delayed: *P* > 0.186; Fig. 4B and C). The same analysis pipeline was conducted at the source level with cluster-based non-parametric permutation tests (alpha = 0.05 and 1,000 permutations, k ≥ 30) averaged over the peak periods identified at the sensor level (P250 for short: 200 to 274 ms). When deviance occurred in the short early condition, deviance detection-related activity was observed in a somatosensory-motor network similar to the generators observed for the pre-deviant standards (Fig. 4A).

Lastly, we investigated the deviance-related changes in directional connectivity between the regions that participated in deviance detection in each condition using Granger causality NxN analysis as implemented in Brainstorm (Tadel et al. 2011). Contrasts of the Granger causality matrices of deviants and standards (Fig. 4A) were performed using paired Wilcoxon tests (1,000 permutations, alpha = 0.05) and an FDR correction was applied (Fig. 4B). No significant effects were observed for both long conditions (early and delayed) and the short delayed condition. For the short early condition, deviance detection was associated with increasing GC values from the left SMA toward the left motor cortex (*P* = 0.004). Note that without FDR correction, and an alpha of 0.01, connectivity results are observed for the long delayed condition. The left SMA (*P* = 0.007), right SI (*P* = 0.007) and right secondary auditory cortex (*P* = 0.008) send information toward the left IFG. With these more liberal parameters, the short early condition and long early condition connectivity results remain the same.

## Discussion

The objective of our experiment was to (i) identify the electrophysiological markers and brain regions involved in the processing of tactile temporal deviance, (ii) investigate the generators of these components, and (iii) clarify the debate surrounding the central clock models and distributed sensory-specific models of temporal perception by considering the length of to-be-encoded temporal intervals. We first identified 2 distinct electrophysiological components, the P250 for the short early deviance detection and the N400 or FN400 for the long condition deviance detection. Then, using source reconstructions, we demonstrated the involvement of a primarily automatic/sensory/motor network for the short early condition, and of a central-executive network for the long conditions. These results suggest that, in line with our recent studies (Thibault et al. 2023; Thibault et al. 2024) in the auditory modality, short intervals rely primarily on modality-specific processes while longer intervals recruit a more distributed network. We used 1.2 s as the boundary in our current experiment, which is consistent with prior work (Grondin 2014, 2012). There is however, variability in this threshold across the timing literature which suggests it is influenced by factors such as task design, leading to a vast proportion of the literature referencing short and long timing as sub- and supra-second, respectively (Lewis and Miall 2003b). Notably, Lewis and Miall (2003a) compared 0.6 s and 3 s intervals while others such as Hellström and Rammsayer (2004) compared 50 ms to 1,000 ms intervals. There have been considerable psychophysical studies that have documented the distinctions of sub-second and supra-second timing to some extent such as Karmarkar and Buonomano (2007) who have found that for short intervals, temporal information may be encoded in the local neural dynamics, aligning with our modality-specific short interval results. Rammsayer et al. (2015) have also identified a transition of modality-specific timing for short intervals to a more cognitive process of time perception with supra-second intervals, which is similar to the results of our current experiment.

### Somatosensory sERP

Standard somatosensory ERPs (sERPs) were thoroughly identified to ensure good data quality (Fig. 1). We observed classic frontocentral P100 and P200 components in response to the vibrotactile stimuli, with activity centered around fronto-central and centro-parietal electrodes (Hoechstetter et al. 2001; Morozova et al. 2023) with generators in bilateral primary somatosensory cortices (Francis et al. 2000; Kim et al. 2016).

### Absence of the CNV in time perception processes using tactile stimuli

The CNV has typically been identified as a marker of temporal accumulation (Macar and Vidal 2003; Kononowicz and Penney 2016; Thibault et al. 2023). However, using the same paradigm as Thibault et al. (2023), but only changing the sensory modality, we did not observe the CNV for any condition in the present experiment. We have recently demonstrated that musical expertise allows to bypass the typical timing-related CNV in the auditory modality (Thibault et al. 2024). Here, we show that in the tactile modality, in non-musicians, temporal accumulation may be processed differently than in the auditory modality. Contrary to previous results (see Macar and Vidal (2004) for a review), our results suggest that the CNV may not be a marker for temporal accumulation in the tactile modality as it was for long auditory intervals in Thibault et al. (2023). More studies are required to investigate the temporal accumulation in tactile conditions.

### Distinct electrophysiological markers supporting deviance detection for short and long intervals processing for tactile stimuli

Our findings concerning the differences in ERPs for short- and long-time intervals support the idea of distinct processes for deviance detection for empty time intervals that are length specific (Lewis and Miall 2003b). Significant differences between deviants and standards were observed for the short early condition around the 250-ms time window (*P* = 0.02, for 224 to 276 ms, Fig. 2A). Such component has already been identified for the detection of novel tactile stimuli, and for consciously perceived stimulus (Yamaguchi and Knight 1991; Auksztulewicz et al. 2012). The sensory gating hypothesis is another role for the P200-like component. In this case, the P200 may act as a precursor for the allocation of attention to new stimulus, filtering non-relevant information (Näätänen 1992; Chien et al. 2019; Thibault et al. 2023). This sensory gating hypothesis suggests that, in the short timing condition, the P200-like component acts as a sensory precursor for allocating attention to changes within a sensory modality, and thus for deviance detection (Chien et al. 2019). In our paradigm, we propose that deviance elicit such a sensory-gating P200 over the primary somatosensory cortices which serves to alert the system that a change occurred, allocating attention toward it. The P200 is thus a pre-attention component which appears to be modality specific. This suggests that the short timing processes are embedded in the sensory modality from which the stimuli are presented.

A key finding of the current paper lies in the difference between short timing processes in the tactile modality and our previous paper results concerning the processes of short timing in the auditory modality (see Thibault et al. 2023). The processing of such short timing intervals seems to be modality-dependent. In such a scenario, state-dependent models (Hass and Durstewitz 2016) of time perception appear more relevant in the description of this phenomenon. State-dependent timing models have already been identified with short, sub-second, time perception tasks (Buonomano et al. 2009). The state dependent models propose that temporal processing is intrinsically encoded in the state of a neural network, which in our case, would be a sensory network (Buonomano et al. 2009; Goel and Buonomano 2014). For example, in the tactile modality, recurrent activity in the somatosensory region may naturally give rise to a “population clock”, where the state of this neural population serves as a temporal representation in which deviance detection may be elicited by a P250 (as it was in our case). This intrinsic approach aligns with our observation of modality-specificity activity for short time intervals, as the somatosensory cortex’s temporal dynamics appear to directly encode and process these durations as was the case for the auditory cortex when presenting auditory stimuli in our previous experiment (Thibault et al. 2023).

In the long condition, we observe a late N400 component which was either observed over frontal electrodes (delayed condition) or centro-parietal electrodes (early condition). The N400 has more thoroughly been studied in the language literature (Kutas and Federmeier 2011) and has been typically associated with memory processes of semantic and episodic information, including for vibrotactile material (Mecklinger 1998; Guillem et al. 1999; Chow et al. 2007). This functional role of the N400 in our context of deviance detection suggests that long timing in the tactile modality would require a more cognitively controlled network at the expense of sensory processing. Even though the N400 component for early and delayed deviance are very similar in latency and amplitude, their generators differ. The frontal N400 (observed in the long delayed condition), more commonly known as the FN400, seems to be slightly distinct from the typical N400 (Stróżak et al. 2021). Indeed, Strozak et al. (Stróżak et al. 2021) have showed that the FN400 can be a marker of high confidence detection, while the N400 (observed in the early long condition) could be rather elicited for more demanding trials. Furthermore, in lines with the pacemaker-counter model of time perception, delayed vibrotactile stimulus may allow for further accumulation of time units (*pulses*) in the temporal accumulator that leads to easier and more confident deviance detection as compared to the before condition. Moreover, longer intervals are typically harder to discriminate and reproduce than shorter intervals (Grondin 2012). This may explain why we observe a FN400 for delayed deviance while earlier deviants exhibit the centro-parietal N400.

More recently, there has been new evidence suggesting that the N400 may act as a neural correlate of predictive coding (Eddine et al. 2022; Eddine et al. 2024). Predictive coding refers to a computational architecture that is biologically plausible. It features a specific arrangement of feed-forward and feedback connections which implements an optimization algorithm that approximates perceptual inferences, allowing for the prediction of upcoming events (Eddine et al. 2024). Our data are consistent with the presence of such a predictive-coding N400 component for deviance detection of delayed time intervals. However, studies investigating its role in predictive coding are currently restricted in the auditory/language domain. We hypothesize that the N400 may be important for our brain to make predictions, but more studies outside the field of language are necessary. Either way, the N400 is a marker of more advanced cognitive processes supporting the idea of the predominance of cognitive processes during long time perception where the temporal material is much less embedded in sensory processes (Lewis and Miall 2003a, 2003b).

Another hypothesis lies within the dynamic attending theory (DAT) which posits that time perception is linked to rhythmic attending, where attentional oscillations align with external rhythms to optimize temporal predictions (Large and Jones 1999). Unlike SET, which relies on scalar properties and accumulation, DAT explains time perception as a dynamic interaction between attention and rhythmic stimuli such as the ones in our experiment. In the context of our findings, because longer timing requires attentional and cognitive resources, the distinct neural responses observed for supra-second intervals could reflect the engagement of attentional systems in alignment with DAT. Specifically for longer intervals, the activation of executive networks aligns with DAT’s proposition that attention to unpredictable longer intervals requires higher-order cognitive functions (Henry and Herrmann 2014). Furthermore, the results observed in the long interval condition can also be interpreted with the trace theory of time perception (TToP; Killeen and Grondin 2022). According to TToP, the perception of intervals relies on the gradual decay of the initial sensory signal marking an interval. The second sensory signal marking the end of an interval provides a reminder of the initial signal. With the second signal occurring much later at 1.6 than at 0.8 sec, the comparison with the initial signal is weakened and more cognitive processes are necessary. Our findings suggesting that memory processes play a more critical role in the timing of long intervals. The memory-related N400 likely reflects the retrieval or updating of this trace that fades over time.

Anyhow, the observed N400 component for long intervals plays a key role in deviance detection for timing intervals. Traditionally associated with semantic processing and memory retrieval, the N400 has also been implicated in predictive coding frameworks, where it reflects the detection of mismatches between anticipated and deviant sensory inputs (Chow et al. 2007; Eddine et al. 2024). The N400 likely represents the involvement of cognitive networks, such as those involved in working memory and prediction error resolution, relevant for timing-related deviance detection.

Although ERPs offer valuable insights into neural processing, a more detailed understanding of the underlying neural generators, achievable through source reconstructions, can clarify the specific brain regions engaged in these processes. Identifying these neural generators is essential to inferring the cognitive and perceptual mechanisms underlying the processing of both short and long-time intervals.

### Different networks underlying short and long timing processes

We identified the PMC and the motor cortex as generators for the P250 component in the short early condition and N400 and FN400 components in the long conditions. The PMC has been acknowledged to play an important role in beat-based timing (Mendoza and Merchant 2014; Penhune and Zatorre 2019). Experiments using intracerebral recordings in the macaque demonstrate that certain neurons of the PMC appear to synchronize to a presented rhythm (Merchant et al. 2013). Such studies were replicated with non-invasive techniques for the human brain (Chen et al. 2008; Pollok et al. 2017). The motor cortex activation coupled with motor preparation processes illustrated by the PMC is also typical for beat and time perception (Mendoza and Merchant 2014; Merchant et al. 2015; Merchant and Averbeck 2017). Thus, these brain regions may be necessary for temporal tactile deviance detection independently of the interval length. However, we have identified regions of the brain which are unique to either short and long interval processing.

#### Short Intervals

As depicted in Fig. 3A, the P250 component observed between 235–245 ms after stimulus onset in the short early condition originates only from the left primary SI, motor cortex, SMA, and PMC. The implication of the SI in the timing of short intervals and marked with tactile stimuli was expected and suggest sensory-specificity for the processing of short timing. The SMA has been reliably recognized as the temporal accumulator in timing tasks (Macar et al. 2002; Macar et al. 2006; Casini and Vidal 2011; Schwartze et al. 2012; Coull et al. 2015; Thibault et al. 2023). This would have supported the hypothesis if we had observed it exclusively in the delayed conditions, however it is not the case in the present study. We propose that in the present experiment, the SMA activation in the short condition may reflect tactile sensory-attention (Legon et al. 2013) and intention-to-act motor processes (Goldberg 1985), commonly observed during the coactivation of the SMA and the motor cortex during such processes (Cunnington et al. 2002; Shibasaki and Hallett 2006; Côté et al. 2020). Connectivity analyses were only significant for the short-early condition. The results show where connectivity, measured by Granger Causality values, is stronger for deviant intervals compared to standard time intervals. It isolates the network that is necessary for deviance detection in the short early timing condition. The result highlights a network where the SMA sends information to the motor cortex, which is in line with our hypothesis of motor preparation processes for short timing intervals. Overall, these findings support the theory stipulating that the processing of short time intervals is based on the contribution of an automatic/sensory/motor (Lewis and Miall 2003b) neural network and is modality-specific.

#### Long Intervals

For longer intervals, a much more distributed network is recruited for the F/N400 component. In both early and delayed conditions, the timing process involves the IFG, the motor cortex, the temporal pole, the insula, and the PMC. More specifically, in our case, longer time intervals, which are harder to discriminate than shorter intervals (Grondin 2012), require the IFG for the processing of deviance. The IFG is well documented as a key structure for the maintenance and manipulation of somatosensory input (Spitzer et al. 2010; Spitzer et al. 2014; Schmidt et al. 2021), especially for more difficult deviance detection (Doeller et al. 2003). It has also been thoroughly identified as a hub of deviance detection which requires higher cognitive contribution (Doeller et al. 2003; Rinne et al. 2005; Tse and Penney 2008). Both the delayed and early long conditions also elicit activation of the insula. The insula has been identified to play a key role in how we integrate bodily signals to make sense of the passage of time, especially for longer time ranges (Craig 2009; Wittmann et al. 2010; Wittmann and Meissner 2019). The insula has also been identified as an attentional hub concomitant with central executive network activation in generating accurate responses to salient events (Menon and Uddin 2010). The motor cortex’s involvement has been controversial in the short vs. long timing literature, with some studies showing its activation in short timing (Lewis and Miall 2003a; Kilavik et al. 2014), others in long timing (Thibault et al. 2023). We postulate that the motor cortex’s implication in the long timing range is less related to classical motor processes, but more to beat-keeping. Just like using explicit counting is more efficient for longer than shorter intervals (Grondin et al. 1999; Grondin et al. 2004), certain beat keeping strategies, that do not require movement, could also be more efficient for longer intervals. Deviance detection in the temporal pole can be associated somatosensory memory (Liu et al. 2016), embedded in higher cognitive functions, reflecting that longer time intervals require higher cognitive processes.

In the long conditions, there are also structures which are unique to either the early or delayed deviants. The following structures may be related to the processes related to longer interval timing but may also be related to whether they elicit surprise (early) or allow for extra pulse accumulation (delayed). Notably, in the long early condition, activation of the PFC is observed, which is consistent with an effect referred to as the “effect of surprise” occurring when a stimulus arrives earlier than expected (Wessel and Huber 2019). In our experiment, earlier deviants typically create this effect of surprise for our participants, who are not expecting the vibrotactile stimulus so soon. Moreover, it has been shown to play a role in relevancy-based gating, inhibiting responses to irrelevant stimulus (Grunwald et al. 2003; Adams et al. 2019), in line with a more cognitive-oriented process of longer temporal intervals. In the delayed condition, we observed the SMA, which has been consistently identified as the temporal accumulator in timing tasks (Macar et al. 2002; Macar et al. 2006; Casini and Vidal 2011; Schwartze et al. 2012; Coull et al. 2015; Thibault et al. 2023). It is thus not surprising to be observing its activation in the delayed deviant condition, where the brain accumulates more time units (*pulses*) than in the standard condition. The extra pulses accumulated in the SMA from the SI, may then be transferred into an auditory code via the secondary auditory cortex (Mioni et al. 2016) for higher-order cognitive processing. Inferior parietal lobe activation is also observed in time perception tasks in the longer duration ranges (Lewis and Miall 2003a). The parietal regions have been documented in the orientation of attention and working memory (Klingberg et al. 1997; Alain et al. 2008; Davranche et al. 2011). The parietal cortex has also been identified to play a key role in the accumulation preceding decision-making, which would be in line with the accumulation of pulses in the delayed condition (FitzGerald et al. 2015; Scott et al. 2017; Zhang et al. 2022). These results are also in line with the hypothesis stipulating that the processing of longer intervals requires a more distributed and cognitive network and are much less modality specific.

The connectivity results for the long delayed condition were non-significant. However, without FDR correction and an alpha of 0.01, we observe that the SMA, the SI, and the secondary auditory cortex send information to the IFG. This would further support our hypothesis of the modality non-specificity in the processing of longer time intervals. This finding provides additional evidence that, as previously discussed, sensory regions interact to transfer timing information stored in the SMA and SI to the secondary auditory cortex, translating it into an auditory code (Mioni et al. 2016). Subsequently, higher cognitive functions, such as working memory and complex deviance detection involving the IFG, are engaged. Although strictly speaking, this result is not statistically significant (with FDR correction), it perfectly illustrates the directionality of the source reconstruction results.

### Post-deviant standards

To investigate the potential updating of the memory trace over time, we performed the contrast between deviants and standards presented immediately after deviants (post-deviant standards). Based on the model-adjustment hypothesis for oddball paradigms (Winkler 2007; Garrido et al. 2009; Winkler and Schröger 2015) we expected that post-deviant standards should involve a re-updating of the memory trace, and thus reflect on cognitive processes. Along these lines, the deviance related N400 component identified in Fig. 2 should be similar between deviants and post deviant standards. In other words, the contrast between deviants and post-deviant standards should not be significant for the N400 time period. As expected, when contrasting the post-deviant standards to the deviants, the results showed no significant differences in the long timing condition across all time periods (early: *P* > 0.172; delayed: *P* > 0.186; Fig. 4B and C). This observation aligns with our predictive coding and the memory mechanisms hypotheses associated with the N400, as observed in our contrasts between pre-deviant standards and deviants (Fig. 2).

In contrast, short timing conditions appear to rely on different mechanisms that are less dependent on cognitive processes, and more on sensory-specific processes. We replicated the early P250 component (deviants vs. post-deviant standard contrast, 200–274 ms, *P* = 0.034; Fig. 4A) we observed with pre-deviant standards (Fig. 2A). The source generators were also very similar, located within a somatosensory-motor network. The consistency of these results highlights that the timing of short intervals is modality-specific while long timing is not.

## Conclusion

In this study, we first identified a pre-attentional P250 for the processing of short early intervals while the long intervals exhibited a tactile-memory-related N400 or FN400. Then, using source reconstructions, we demonstrated the involvement of a primarily automatic/sensory/motor network in the short early condition and of a primarily central-executive network for the long conditions. In accordance with our past studies (Thibault et al. 2023; Thibault et al. 2024), we demonstrate that shorter intervals rely primarily on modality-specific processes while longer intervals recruit a more distributed network.

## Data Availability

Data and pipelines for analyses will be available via Université Laval’s repository link upon acceptance: https://doi.org/10.5683/SP3/V5102U.
